# Unraveling immunotherapy's role in propelling cancer outcomes

**DOI:** 10.1002/cnr2.1862

**Published:** 2023-07-06

**Authors:** Ning Ji, Amit K. Tiwari, Dong‐Hua Yang

**Affiliations:** ^1^ Tianjin Medical University Cancer Institute and Hospital National Clinical Research Center for Cancer, Tianjin's Clinical Research Center for Cancer, Key Laboratory of Cancer Prevention and Therapy Tianjin China; ^2^ College of Pharmacy University of Arkansas for Medical Sciences Arkansas USA; ^3^ Clinical Medicine New York College of Traditional Chinese Medicine Mineola New York USA

Immunotherapy is widely accepted as an effective treatment for various solid and hematologic cancers, which is an important advancement in cancer research. Using immunotherapeutic principles, a series of drugs and strategies have been developed to improve the prognosis of specific cancers and other malignancies. However, immunotherapy's efficacy and the formidable challenge posed by drug resistance are notable limitations to its widespread application in clinical settings. These challenges are compounded by the lack of specific biomarkers that can accurately predict immunotherapy's outcome. These critical issues are addressed in this special issue topic, which includes exemplary studies that explore current obstacles and future possibilities in immunotherapy. In addition to examining both fundamental research and clinical application, these studies provide valuable insights to the cancer research community, ensuring their relevance and significance as we advance our understanding of this transformative field.

In a study by Difoum et al.,^1^ it was discovered that several factors, including female sex, primitive tumor SUVmax<5, the presence of ≥3 metastases, and receiving immunotherapy after the first line of treatment, were identified as significant risk factors for immune‐related adverse events. These findings highlight the potential utility of the ToxImmune score in clinical practice, enabling personalized toxicity surveillance for patients undergoing immunotherapy for lung cancer.

According to the review article by Huss et al.,^2^ digital biomarkers and artificial intelligence are potential tools for guiding immune therapy selection and advancing precision oncology. This underscores the importance of incorporating digital and computational approaches, alongside standardized strategies in histopathology, and the use of mathematical tools to aid clinical and diagnostic decision‐making as fundamental principles in the realm of “precision oncology”. Through precision pathology and artificial intelligence, the study emphasizes the importance of guiding immune therapy selection and advancing precision oncology.

The use of immunotherapy in combination with other treatments can be very effective. For patients with recurrent head and neck mucosal melanoma, Musha et al.^3^ examined whether immune checkpoint inhibitors (ICIs) like nivolumab, pembrolizumab, and ipilimumab were effective when combined with carbon‐ion radiotherapy. The results of the study showed that the combination therapy was safe and effective. Mahase et al.^4^ studied the use of concurrent immunotherapy and stereotactic body radiotherapy to treat recurrent high‐grade gliomas successfully. Combining ICIs with radiotherapy provides valuable insights into how immunotherapy can be used in combination with other treatments in clinical settings.

The reintroduction of immune checkpoint inhibitors (ICI) after severe immune‐related adverse events (irAEs) remains a subject of debate. In a case report by Nair and Trehan,^5^ a 65‐year‐old male with recurrent and metastatic sinonasal squamous cell carcinoma (R/M‐SNSCC), who had achieved remission with pembrolizumab monotherapy, experienced high‐grade pneumonitis during treatment, leading to ICI discontinuation. Pembrolizumab was successfully tolerated by the patient for 10 months, resulting in a stable disease state. It appears that resuming ICI at a lower dose while concomitantly suppressing the immune system may be a desirable approach following high‐grade checkpoint‐inhibitor pneumonitis (CIP).

In the management of advanced esophageal squamous cell carcinoma (ESCC), immunotherapy has emerged as a viable option. Based on the results of a meta‐analysis conducted by Jin et al.,^6^ the clinical outcomes of PD‐1/PD‐L1‐based immunochemotherapy were compared with chemotherapy alone in advanced ESCC. It was found that PD‐1/PD‐L1 immunochemotherapy significantly enhanced survival outcomes for patients with advanced ESCC. According to Takehara et al.,^7^ in a case study, combining immune checkpoint inhibitors (ICIs) with radiotherapy shows promise for patients with immune checkpoint‐inhibitor‐resistant ESCC. The results of this study support those of Musha et al.,^3^ further demonstrating the potential effectiveness of ICIs combined with radiotherapy in managing ESCC. For advanced esophageal squamous cell carcinoma patients, immunotherapy, specifically PD‐1/PD‐L1‐based treatment combined with radiotherapy, is shown to have a significant impact on patient outcomes.

Overall, immunotherapy has proven efficacious in various tumor types, but its widespread clinical application faces a number of challenges. It is necessary to develop novel agents and approaches in order to address the intricate interplay between the immune system and tumor heterogeneity. For optimal efficacy and safety, radiotherapy and targeted treatments must be integrated. The importance of personalized precision therapy, which takes into account factors such as gender, age, immune cell ratio, and molecular markers, cannot be overstated. To understand how these factors impact treatment effectiveness, safety, prognosis, and side effects, comprehensive research is needed. It is only through advancing immunotherapy knowledge and tailoring interventions that we will be able to unlock its full potential and revolutionize cancer treatment.
